# Damping Behaviour and Mechanical Properties of Restorative Materials for Primary Teeth

**DOI:** 10.3390/ma15217698

**Published:** 2022-11-02

**Authors:** Thomas Niem, Roland Frankenberger, Stefanie Amend, Bernd Wöstmann, Norbert Krämer

**Affiliations:** 1 Department of Prosthodontics, Medical Center for Dentistry, Justus Liebig University Giessen and University Hospital Giessen and Marburg, Campus Giessen, Schlangenzahl 14, 35392 Giessen, Germany; 2 Department of Operative Dentistry, Endodontics and Pediatric Dentistry, Medical Center for Dentistry, University of Marburg and University Hospital Giessen and Marburg, Campus Marburg, Georg Voigt Strasse 3, 35039 Marburg, Germany; 3 Department of Paediatric Dentistry, Medical Centre for Dentistry, Justus Liebig University Giessen and University Hospital Giessen and Marburg, Campus Giessen, Schlangenzahl 14, 35392 Giessen, Germany

**Keywords:** modulus of toughness, Leeb hardness, Martens hardness, Vickers hardness, elastic deformation, plastic deformation, viscoelastic properties, damping, energy dissipation, deciduous teeth

## Abstract

The energy dissipation capacity and damping ability of restorative materials used to restore deciduous teeth were assessed compared to common mechanical properties. Mechanical properties (flexural strength, modulus of elasticity, modulus of toughness) for Compoglass F, Dyract eXtra, SDR flow, Tetric Evo Ceram, Tetric Evo Ceram Bulk Fill, and Venus Diamond were determined using a 4-point bending test. Vickers hardness and Martens hardness, together with its plastic index (η_ITdis_), were recorded using instrumented indentation testing. Leeb hardness (HLD) and its deduced energy dissipation data (HLD_dis_) were likewise determined. The reliability of materials was assessed using Weibull analysis. For common mechanical properties, Venus Diamond always exhibited the significantly highest results and SDR flow the lowest, except for flexural strength. Independently determined damping parameters (modulus of toughness, HLD_dis_, η_ITdis_) invariably disclosed the highest values for SDR flow. Composite materials, including SDR flow, showed markedly higher reliabilities (Weibull modulus) than Compoglass F and Dyract eXtra. SDR flow showed pronounced energy dissipation and damping characteristics, making it the most promising material for a biomimetic restoration of viscoelastic dentin structures in deciduous teeth. Future developments in composite technology should implement improved resin structures that facilitate damping effects in artificial restorative materials.

## 1. Introduction

Extremely stable and shock-absorbing natural composite materials, which may be found for example in bone and tooth structures too [1], show the common feature of allowing a harmless and repeated dissipation of dangerous impact load, which in unfavourable conditions could be the reason to initiate destruction processes. Precisely characterising the physical properties and physicochemical mechanisms operating in the background during stress impact is therefore crucial so that structures can be effectively protected from disruptive effects. This mimics what the natural periodontal ligament in pristine tooth structures does with its viscoelastic damping properties by similarly connecting rigid tooth and bone structures via tough protein tissues [2,3]. Characterising damping properties is also important to better understand the various behaviours of artificial restorative materials. Then these can be used to replace the damping effects of naturally occurring resilient structures in a manner that is as biomimetic as possible.

If this goal is to be achieved, due consideration must be given to the fact that enamel and dentin of the natural tooth substrate exhibit different mechanical characteristics [4] and that primary dentition may demonstrate a deviant behaviour during load impact compared to permanent dentition [5]. Furthermore, it was recently reported that deciduous dentin showed markedly higher loss tangent data, and with that pronounced damping characteristics, than the respective structures of permanent teeth [6]. This may be unequivocally attributed to higher viscoelastic material behaviour. Generally, dentin structures appear to have superior viscoelastic material properties and accordingly pronounced chances to dissipate fracture energy [7,8]. These objective facts should therefore be taken into account when specific restorative materials are selected in clinical practice, especially for deciduous teeth.

Next, the question arises as to whether commonly determined mechanical properties such as flexural strength, Young’s modulus, fracture toughness, surface hardness, and abrasion resistance [9] adequately allow a selection of suitable restorative materials with appropriate energy dissipation effects and damping characteristics. We recently reported three useful methods to assess the damping properties of computer-aided design and computer-aided manufacturing (CAD/CAM) materials where the modulus of toughness (MT) values [10], Leeb hardness data (HLD) together with its deduced energy dissipation results (HLD_dis_) [11,12,13], as well as the instrumented indentation testing (IIT) together with its elastic (η_IT_) and plastic (η_ITdis_) index [11] were determined. All three methods are highly suitable for damping assessment in the present investigation.

Since force absorption capabilities and the presence of energy dissipation and damping characteristics are highly appreciated in clinical practice, also when restoring deciduous teeth, the identification and reliable determination of such effects is an important prerequisite to allow a reasonable comparison of different restorative materials. At present, little meaningful information is available regarding these properties. Therefore, the aim of this in vitro study was to determine the energy dissipation and damping characteristics of restorative materials commonly used for deciduous teeth by determining MT, HLD_dis_, and η_ITdis_ data and comparing the results with common mechanical properties like FS, ME, HM, HV 1 and Weibull parameters. The null hypothesis tested was that the damping parameters (MT, HLD_dis_, and η_ITdis_) investigated are independent of the restorative material tested.

## 2. Materials and Methods

### 2.1. Specimen Preparation and Storage Conditions

For the present comparative study, six commonly used restorative materials were selected from the different material types, compomers and composites (Table 1). While dental composites usually consist of a resin mixture (app. 25–30 wt%), different filler types (app. 70–75 wt%) and additives (app. 1–5 wt%) [14], compomer restorative materials additionally contain polymeric acrylic acid derivatives in combination with calcium-aluminium-silicate glasses, which may react in the presence of water like classic glass-ionomer materials do with the formation of cement structures [14]. All materials were stored, used and handled under ambient laboratory conditions (23 ± 1 °C; 50 ± 5% relative humidity).

For the bending test, twenty specimens were prepared for each restorative material using a stainless-steel mould (2.0 ± 0.1 mm × 2.0 ± 0.1 mm × 25.0 ± 2.0 mm) according to ISO 4049 [18]. Curing was performed on the top and bottom of the respective specimens using a LED curing unit (SmartLite PS, Dentsply DeTrey GmbH, Konstanz, Germany) with several overlapping light exposures (no more than 1 mm of the diameter of the light guide) and not less than 10 s per exposure, but in accordance with the user instruction of the respective restorative material. After removal from the mould, specimen dimensions were adjusted, if necessary, by grinding on wet silicon carbide paper (grit size P 1200, Leco Corp, St. Joseph, MI, USA).

For Leeb hardness determination and IIT testing, two disc-shaped specimens were prepared for each restorative material using a stainless-steel mould (diameter: 20.0 ± 1.0 mm, height: 3.5 ± 0.1 mm). The restorative materials were pressed between two glass plates on the top and bottom of the mould with polyester sheets (50 µm thickness, Hostaphan RN 50, Mitsubishi Polyester Film GmbH, Wiesbaden, Germany) in between the material and glass. Polymerisation was performed in a curing oven (Uni XS, Heraeus Kulzer GmbH, Hanau, Germany) from both sides with 180 s applied to each side. The curing oven was used to achieve a homogenous curing of the whole specimen, thus avoiding overlapping effects that occur using a hand polymerisation lamp. After removal from the mould, specimen dimensions were adjusted, if necessary, by grinding on wet silicon carbide paper (grit size P 1200, Leco Corp, St. Joseph, MI, USA) and polished (grit size P 4000, Leco Corp, St. Joseph, MI, USA). Special care was taken to generate absolutely flat surfaces on one side of the specimen without any rims or fringes to allow an even positioning on the reference block for Leeb hardness determination [19].

All specimens used for the 4-point bending test were stored in distilled water (Aqua B. Braun, Melsungen, Germany) using separate glass vessels that were placed in an incubator (Ehret, Emmendingen, Germany) at 37 °C for 24 h. A more reliable material comparison was achieved by storing specimens intended for hardness determination for 24 h at ambient laboratory conditions (23 ± 1 °C; 50 ± 5% relative humidity) without water contact to avoid plasticising effects induced by water, which could have influenced the viscoelastic and plastic material properties as previously described [20].

### 2.2. Test Procedures and Material Investigation

FS, ME, and MT were determined using the 4-point bending test (*n* = 20) [18]. After water storage, specimens were placed for one minute at ambient laboratory conditions on a tissue paper for drying and subsequently fixed between four support bars (Ø = 2.0 mm; distance of inner/upper support bars: 10.0 mm; distance of outer/lower support bars: 20.0 mm). The specimens were then loaded until fracture with a crosshead speed of 1.0 mm/min [18] in a universal testing machine (Z 2.5, Zwick/Roell, Ulm, Germany) using a 2.5 kN load cell. FS (MPa) [18,21,22,23], ME (MPa) (tangent method with 0.10% plastic deformation) [21,22,23] and MT (MJ/m^3^) [24] were calculated using the following Equations (1)–(3) and the software of the testing machine (TestXpert II release V3.4, Zwick/Roell, Ulm, Germany) based on the respective stress-strain diagrams:

(1) $$FS=\frac{3F_{\max}L}{4wh^{2}}$$

(2) $$ME=\frac{0.17\;mL^{3}}{wh^{3}}$$

(3) $$MT=\frac{9A}{wh\;\left(3L-4a\right)}$$

where *F_max_* (N) is the maximum load, *L* (mm) is the span width between the two outer (lower) support bars, *w* (mm) and *h* (mm) are the width and height of the specimen, *m* (N/mm) is the slope of the tangent to the initial straight line, *A* (J) is the total area under the load-deformation curve (work performed by the applied load to deflect and fracture the specimen), and *a* (mm) is the half of the difference between the lower and upper fixture spans (L/4). The 4-point bending method was used because it concentrates the stress over a wider area of the tested beam [25], in contrast to the 3-point bending mode. This allows the beam to fail from a position directly under the applied load, which is defined as a prerequisite from mechanics’ point of view.

**Figure 1:**
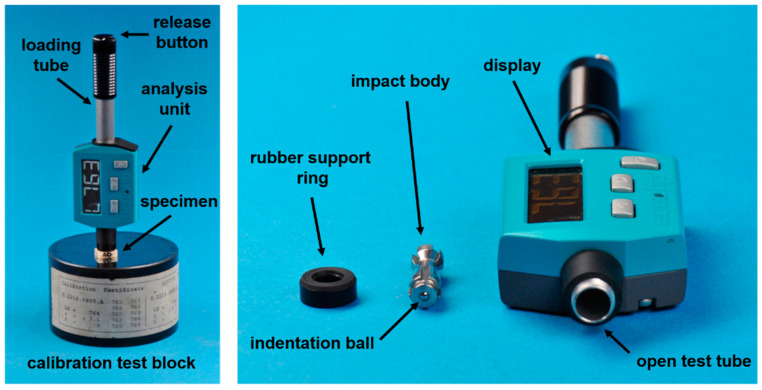
Leeb hardness testing device (Equotip Piccolo 2). Left side: Device placed together with specimen on the calibration test block; right side: disassembled device (from left to right: rubber support ring, impact body with indentation ball; open test tube).

The HLD test device used (Figure 1) was newly calibrated by the manufacturer, who provided a calibration certificate. All indentations were conducted under ambient laboratory conditions (23 ± 1 °C; 50 ± 5% relative humidity) on dry specimen surfaces with the device-specific impact body and its indentation ball (tungsten carbide; 1500 HV) (Figure 1). Before measurements were performed on specimen surfaces, the device accuracy was carefully controlled as recommended by the manufacturer [19] and as previously described [11]. The calculated mean exhibited a maximum deviation of 7 HLD units (0.93%) when compared to the certified value specified on the reference block.

HLD of the restorative materials was unequivocally determined by centrical positioning on the calibration test block, which was placed on a 10 mm thick granite plate. The indentation process was carried out as previously described [11]. Five indentations were performed on the same specimen surface, with the arrangement very closely resembling a five depicted on dice to ensure the required distances specified in the ISO standard [19]. Two specimens were examined for each restorative material to yield ten values in total. The dimensionless HLD values were internally calculated via the device software (4) as previously explained [26] and defined [19] and directly read off from the device display:

(4) $$HLD=\frac{\nu_{R}}{\nu_{A}}\times 1000$$

with *ν_R_* (rebound velocity) in m/s and *ν_A_* (impact velocity, 2.1 m/s). Consequently, the amount of energy that was logically lost (dissipated and converted) on the material surface and not possible to recover was simply deduced from *HLD* by means of subtracting HLD values from 1000 (5). The respective results were defined *HLD_dis_* to describe energy dissipation effects as previously explained [11]:

(5) $$HLD_{dis}=1000-HLD$$

Vickers hardness as well as Martens hardness and its elastic (*η_IT_)* and plastic (*η_ITdis_*) index were determined using a newly calibrated universal hardness-testing machine (ZHU0.2, Zwick Roell, Ulm, Germany) [27] in accordance with ISO 14577-1 [28]. Via software control, the diamond indenter pyramid (*α* = 136°) of the testing machine was pressed vertically into the investigated specimen surface using a crosshead speed of 0.3 mm/min until a load of 10 N was reached, which was held for 10 s before the crosshead was driven back. Force-indentation depth curves were recorded during the entire indentation process with a simultaneous determination of the totally consumed indentation work/energy (*W_total_*) and its elastic (*η_IT_*) and plastic (*η_ITdis_*) energy proportion using the specific software of the testing machine (testXpert V12.2, Zwick Roell, Ulm, Germany). *HM* (N/mm^2^), *η_IT_* (%), and *η_ITdis_* (%) were calculated based on Equations (6)–(8) (ISO 14577–1) [28], and *HV* 1 (in kp/mm^2^) based on Equation (9) (ISO 6507-1) [29]:

(6) $$HM=\frac{F}{A_{S}(h)}$$

(7) $$\eta_{IT}=\frac{W_{elast}}{W_{total}}\times 100$$

(8) $$\eta_{ITdis}=100\%-\eta_{IT}=\frac{W_{plast}}{W_{total}}\times 100$$

(9) $$HV\;1=\frac{0.1891\;F}{d^{2}}$$

with *HM* (Martens hardness) in N/mm^2^, *F* (maximum test load) in N, *A_s_ (h)* (surface area of the indenter at distance h from the tip) in mm^2^, *η_IT_* (elastic to total work ratio) in %, *W_elast_* (recoverable elastic work during indentation) in Nm, *W_total_* (total work during indentation) in Nm, *W_plast_* (non-recoverable plastic deformation work during indentation) in Nm, *η_ITdis_* (plastic to total work ratio) in %, *HV* 1 (Vickers hardness number) in kp/mm^2^, and *d* (average length of both indentation diagonals) in mm. Indentations were conducted under ambient laboratory conditions (23 ± 1 °C; 50 ± 5% relative humidity). For all restorative materials, each specimen (see Section 2.2) was centrically positioned under the indenter head, and five indentations were performed in a row on the same specimen surface. Two specimens were examined for each restorative material.

Surface topographies, indentation volumes and depths were analysed with an optical profilometer (MicroProf 200, Fries Research & Technology GmbH, Bergisch Gladbach, Germany). A 600 µm sensor with a precision of 200 nm and a vertical resolution of 20 nm was used, and the investigated area was 2.50 mm × 2.50 mm in size (sensor frequency 1000 Hz). Surface profiles were optically analysed using Mark III Software (release 3.11.5.2, Fries Research & Technology GmbH, Bergisch Gladbach, Germany), and raw data were treated with a software-specific smoothing function before measurement. Three independent measurements were performed to calculate the means of the indentation volume (10^5^ µm^3^) and indentation depth (µm). This was done after 24 h storage time at ambient laboratory conditions to account for viscoelastic relaxation processes.

The morphological characterisation of selected surface structures was performed using an Amray 1610 Turbo scanning electron microscope (Amray Inc., Bedford, MA, USA) at an accelerating voltage of 10 kV and a magnification of 2000×. For this investigation pieces were selected directly following the 4-point bending test, fixed on aluminium pin stubs using conductive carbon cement (both Plano GmbH, Wetzlar, Germany) and sputtered with gold (Sputter Coater SC502, Polaron, Fisons Instruments GmbH, Mainz-Kastel, Germany). All observations were conducted by one person.

### 2.3. Statistical Analysis

Mean values and standard deviations of all investigated mechanical properties (*FS*, *ME*, *MT*, *HV 1*, *HM*, *HLD_dis_*, *η_ITdis_*) were calculated, and normality of data distribution was tested using Kolmogorov-Smirnov and Shapiro-Wilk tests. Multiple comparisons of different restorative materials were performed using one-way analysis of variance (ANOVA), and post hoc comparisons were executed using a Games-Howell test. The reliability of the investigated restorative materials and the probability (*P_f_(σ)*) of failure at given loading stress was estimated by performing a Weibull distribution function on *FS* (*n* = 20) using Equation (10) where *σ_C_* is the flexural strength (*FS*), *σ*_0_ is a characteristic strength at a fracture probability of *P_f_(σ)* = 63.2%, and *m* is the Weibull modulus [30].

(10) $$P_{f}\left(\sigma \right)=1-\exp\left[-{\left(\frac{\sigma_{C}}{\sigma_{0}}\right)}^{m}\right]$$

The double logarithm of Equation (10) provides Equation (11). By plotting *lnln*(1/(1 − *P_f_*(*σ*))) versus ln *σ_C_*, a straight line results, with the gradient *m*, whereas the intersection with the x-axes gives the logarithm of the characteristic strength *σ*_0_ [30].

(11) $$\ln\;\left[\ln\left(\frac{1}{1-P_{f}\left(\sigma \right)}\right)\right]=m\ln\sigma_{C}-m\ln\sigma_{0}$$

Coefficient of determination (R^2^) was used to evaluate how well *m* fits the straight line of the graph according to simple linear regression [30]. All statistical analyses were carried out using IBM SPSS Statistics for Windows (version 23.0.0.2, IBM World Trade Corp., Armonk, NY, USA) at a significance level of *α* = 0.05. The Weibull plot and the respective linear regression were prepared with Excel (Office 16, Microsoft Corp., Redmond, WA, USA).

## 3. Results

### 3.1. 4-Point Bending Test

**Figure 2:**
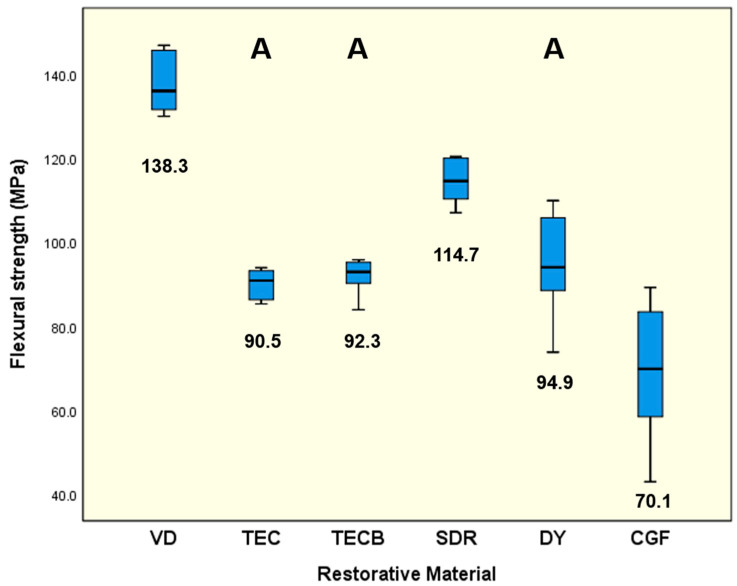
Flexural strength (MPa). Numerical values below each boxplot represent the calculated means. Identical uppercase letters denote no significant difference among calculated means of restorative materials (*p* > 0.05).

**Figure 3:**
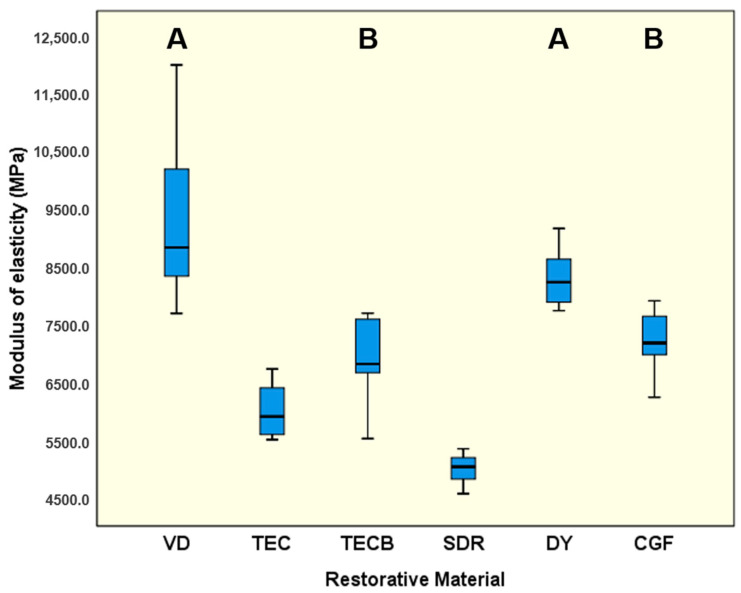
Modulus of elasticity (MPa). Identical uppercase letters denote no significant difference among calculated means of restorative materials (*p* > 0.05) while different uppercase letters represent significant differences (*p* < 0.05).

**Figure 4:**
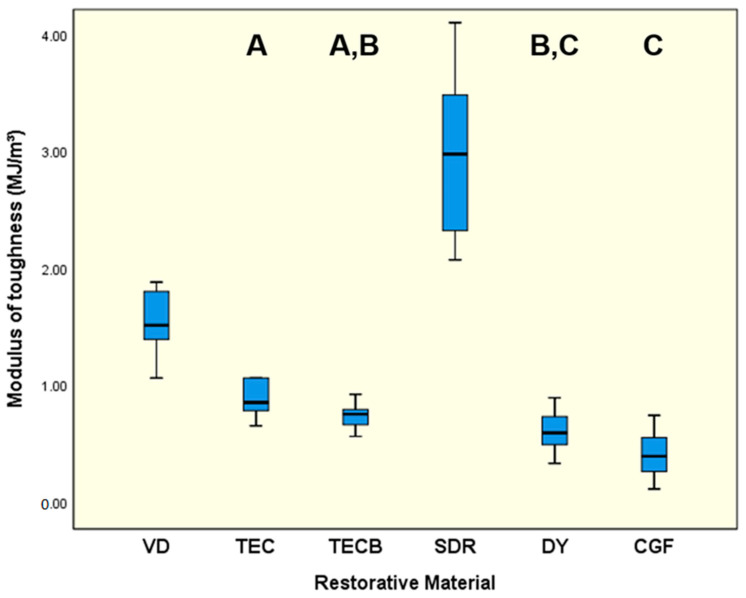
Modulus of toughness (MJ/m^3^). Identical uppercase letters denote no significant difference among calculated means of restorative materials (*p* > 0.05) while different uppercase letters represent significant differences (*p* < 0.05).

Means and standard deviations were calculated, and medians of the parameters obtained from the 4-point bending test (FS, ME, MT) together with the respective statistical results of the material comparisons are displayed in separate box plot diagrams (Figure 2, Figure 3 and Figure 4). All investigated specimens broke during the test procedure. VD showed the highest mean for FS (138.3 MPa) and SDR (114.7 MPa) the second highest data set (*p* < 0.001) (Figure 2). The lowest results were obtained for CGF (70.1 MPa) followed by DY (94.9 MPa), TECB (92.3 MPa) and TEC (90.5 MPa) which all three were statistically not distinguishable (*p* > 0.05) but still significantly higher than CGF (*p* < 0.05).

VD (9220.6 MPa) and DY (8344.0 MPa) were not significantly different (*p* = 0.399), both showed the highest means (*p* < 0.01) for ME (Figure 3), directly followed by CGF (7240.0 MPa) and TECB (6922.3 MPa) which were not significantly different (*p* = 0.83). SDR exhibited the significantly lowest results (5063.4 MPa, *p* < 0.001) followed by TEC with 6034.4 MPa (*p* < 0.01). For MT (Figure 4) SDR unequivocally exhibited the significantly highest results (2.98 MJ/m^3^, *p* < 0.001) followed by VD with 1.55 MJ/m^3^ (*p* < 0.001). The lowest means were obtained for CGF (0.41 MJ/m^3^) and DY (0.62 MJ/m^3^) which were not significantly different (*p* = 0.154).

**Figure 5:**
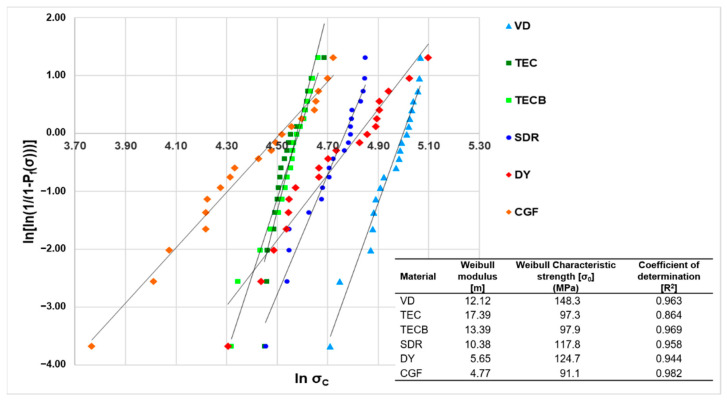
Weibull distribution as a function of material. Weibull modulus m, characteristic strength σ_0_ which is the strength at a probability of failure (P_f_(σ)) of 63.2%, and the coefficient of determination R^2^ which describes the overall fit to the projected linearity.

The modelled Weibull plot is displayed in Figure 5 and the highest material reliability (Weibull modulus m) was found for the composite materials and the lowest results were obtained for compomers. The decreasing order of m is: TEC > TECB > VD > SDR > DY > CGF with very high coefficients of determination (R^2^) being greater than 0.940 except for the results of TEC, where R^2^ was 0.864.

### 3.2. Leeb Hardness

**Figure 6:**
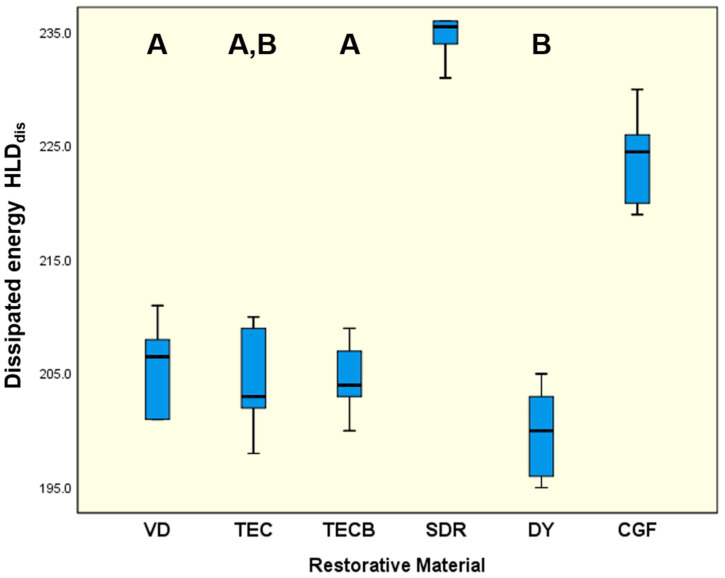
Dissipated energy during Leeb hardness testing (HLD_dis_). Identical uppercase letters denote no significant difference among groups (*p* > 0.05), while different uppercase letters represent significant differences (*p* < 0.05). HLD_dis_ represents a dimensionless property.

**Figure 7:**
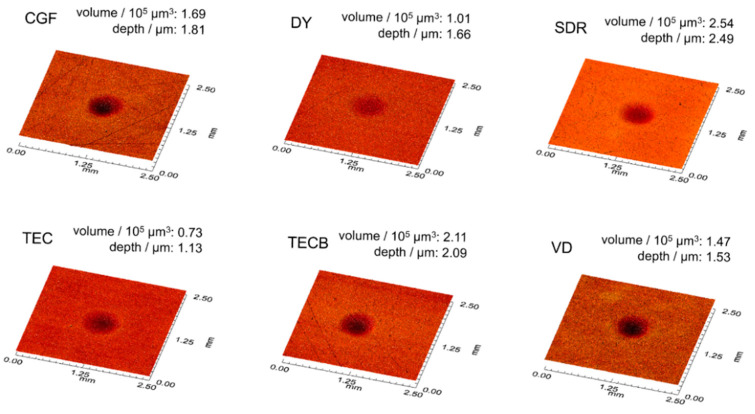
Representative surface topography images of Leeb hardness indentation areas. Indentation volumes and depths are displayed on the respective right upper corner of each image.

For HLD_dis_ (Figure 6) the significantly highest results were unequivocally obtained for SDR (234.8, *p* < 0.001) followed by CGF with 223.8 (*p* < 0.001). The lowest mean was determined for DY (199.8, *p* < 0.05), which was not distinguishable from TEC (204.5, *p* = 0.162). The profilometric evaluation of the respective impact surfaces (Figure 7) showed the deepest indentation for SDR, followed by the other restorative materials in descending order (SDR > TECB > CGF > DY > VD > TEC). The indentation volume and indentation depth data were interpreted merely on a phenomenological basis and not subjected to further statistical analysis, as only three data sets were available per restorative material.

### 3.3. Instrumented Indentation Testing

**Figure 8:**
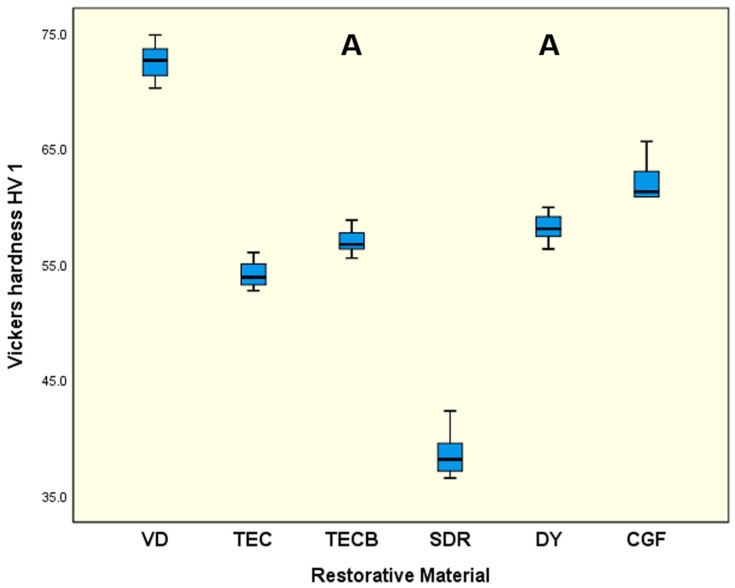
Vickers hardness (HV 1). Identical uppercase letters denote no significant difference among groups (*p* > 0.05).

**Figure 9:**
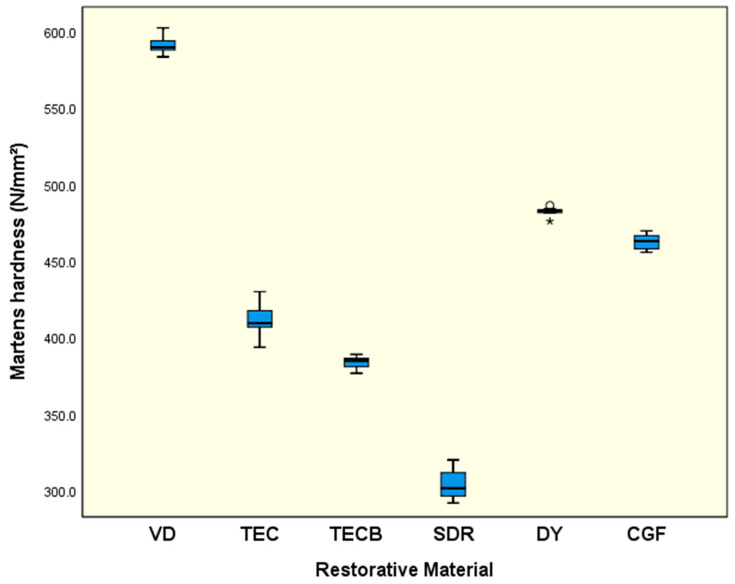
Martens hardness (N/mm^2^). All calculated means are significantly different (*p* < 0.001). Circles represent mild outliers (<3.0 × interquartile range) and asterisks extreme outliers (>3.0 × interquartile range).

For HV 1 (Figure 8) and HM (Figure 9), very similar results were obtained. While VD, in both cases, exhibited the significantly highest means (*p* < 0.001) the lowest results were obtained for SDR (*p* < 0.001). The other restorative materials were ranked in between with DY and CGF tending to exhibit higher data sets than TEC and TECB.

**Figure 10:**
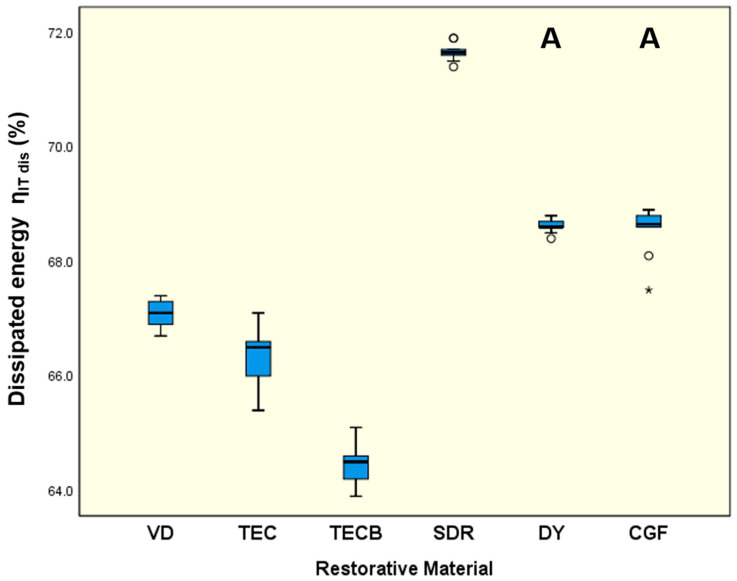
Plastic index of instrumented indentation testing (η_ITdis_) (%). Identical uppercase letters denote no significant difference among groups (*p* > 0.05). Circles represent mild outliers (<3.0 × interquartile range) and asterisks extreme outliers (>3.0 × interquartile range).

For η_ITdis_ (Figure 10) SDR showed the significantly highest results (71.7%, *p* < 0.001) followed by DY (68.6%) and CGF (68.6%), which were not significantly different (*p* = 0.996) but both had significantly higher means (*p* < 0.001) than VD (67.1%), TEC (66.4%) and TECB (64.5%).

## 4. Discussion

### 4.1. Common Mechanical Properties

If the results obtained are interpreted taking just the common mechanical properties into account, then in nearly all cases, VD showed the highest results independent of whether FS and ME were considered (Figure 2 and Figure 3) or whether conventional hardness properties like HV 1 or HM (Figure 8 and Figure 9) were interpreted. Hence, at first glance, VD appeared to be very powerful and the most suitable restorative material.

A detailed comparison of the conventional hardness values determined in the present study in reference to available relevant data from the literature was abandoned as too many data sets investigated had been under different experimental conditions. But the ranking and general trend of very high values obtained for VD and, on the other hand, the lowest results obtained for SDR, with TECB and TEC in between, might be definitively confirmed [31,32]. However, the 4-point bending results obtained can only be compared to a limited extend with similar data in the literature as very little information is available for the materials investigated in the present study. In most studies, 3-point bending tests were instead used to determine mechanical properties (Table 2). Nevertheless, three exceptions were found for 4-point bending. Two were published by Belli et al. [33,34] who obtained FS results for 4-point bending after a 14 d storage time in distilled water. Their disclosed value of 90.6 MPa for TEC [33] (Table 2) perfectly resembles the results obtained in the present study with 90.5 MPa (Figure 2). Similarly, their data set obtained for VD with 130.1 MPa [34] (Table 2) very closely matches the values (138.3 MPa) determined in the present study (Figure 2). This outcome confirms the accurate experimental conditions of the present investigation. The third publication was presented by Suiter et al. [23] who obtained data sets for CGF and DY after storage in distilled water for FS (28 d storage time) and ME (1 d, 7 d, 14 d, and 28 d storage time). Their FS values after 28 d for CGF (67.0 MPa) and DY (78.0 MPa) (Table 2) resemble the results of the present investigation obtained after 1 d (CGF, 70.1 and DY, 94.9) (Figure 2). The slightly higher results, especially for DY, might be due to the markedly shorter storage time in water. Highly surprising results were obtained for ME by Suiter et al. [23]. Although they investigated their samples using the 4-point bending mode, the obtained data after 14 d storage time for CGF (17.4 GPa) and DY (19.9 GPa) were, in both cases, more than twice as high as the respective results of the present investigation (CGF, 7.2 GPa and DY, 8.3 GPa), data from the literature (Table 2) and likewise the available manufacturer’s information (Table 2). Data from the literature and the available manufacturer’s information were divergently determined via 3-point bending tests. The reason for this unexpected deviation is not yet clear, but the special setup with an external deflectometer to determine the displacement and the respective ME calculation could possibly have been the cause. Nevertheless, when comparing the present results of FS and ME to literature data determined via 3-point bending, a good agreement was still observed, showing a general trend of slightly lower values for the present 4-point bending data. This fact of lower FS data for 4-point bending tests compared to 3-point bending had previously been observed for denture-base polymers [35], composites [36,37], and ceramics [38,39], which equally supports the findings of the present study.

### 4.2. Weibull Analysis

When comparing the reliability, Weibull modulus m, with available data from the literature, a perfect match for VD could be found. The respective result of the present study (m = 12.1) (Figure 5) very closely resembles the 4-point bending data after 14 d storage time in water (m = 12.2) published by Belli et al. [34] and the 3-point bending results presented by Graf et al. [43] when specimens were stored 1 d in water (m = 12.9). Unfortunately, for TEC, different results were obtained for the reliability. The present data gave m = 17.4, and the results obtained by Belli et al. [34] gave m = 23.6, which was determined using the same 4-point bending test as described before for VD. The reason for this deviation is not yet clear, but the associated R^2^ value, usually reporting the fit of variance of the observed data towards the projected ideal linearity, was not disclosed by Belli. Nevertheless, the respective value for TEC observed in the present study (R^2^ = 0.864) (Figure 5) was the lowest but still found to be in an acceptable range.

Compared to the present result for SDR (m = 10.4), a Weibull modulus showing values more than twice as high was previously reported (m = 26.7) [46]. This result was obtained with specimens after 24 h storage time in water (37 °C) but divergently by using a 3-point bending test with an extremely reduced span width of solely 12 mm. This markedly reduced span width may explain the deviating results because the volume of a test specimen defines the mean strength, and accordingly, the mean size of the mapped critical flaw [47]. However, this author group published a second article with the same setup under comparable conditions [44] (but different universal testing machines) disclosing a very similar Weibull modulus for SDR as before (m = 26.6) and for TECB a value (m = 11.2) much closer to the results of the present study (m = 13.4). The better match for TECB in comparison to SDR could be due to the use of different polymerisation procedures. Furthermore, for TECB, TEC and SDR, a recent publication [48] similarly reported a Weibull analysis for 3-point bending data (24 h; 37 °C saline solution). Unfortunately, only the Weibull plots were disclosed, and no further details about the Weibull modulus or the characteristic strength were given. Therefore, a more meaningful comparison could not be made.

**Figure 11:**
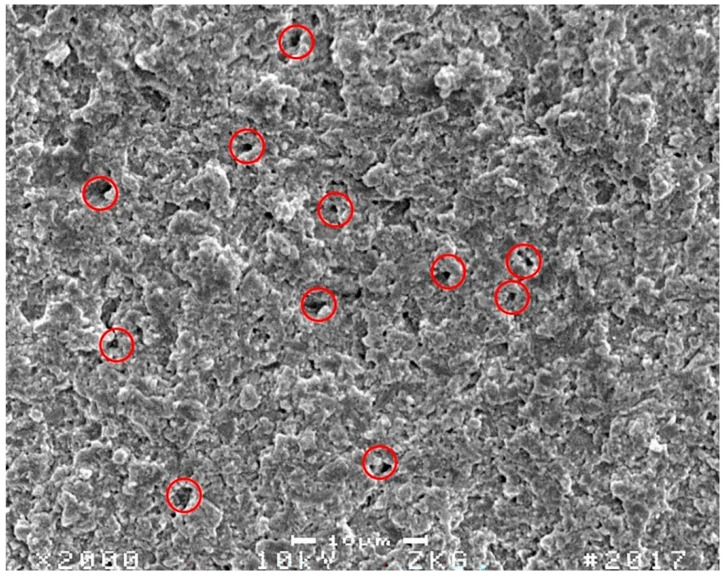
Representative SEM image of a selected CGF fracture surface. Red circles exemplarily denote material inhomogeneities.

**Figure 12:**
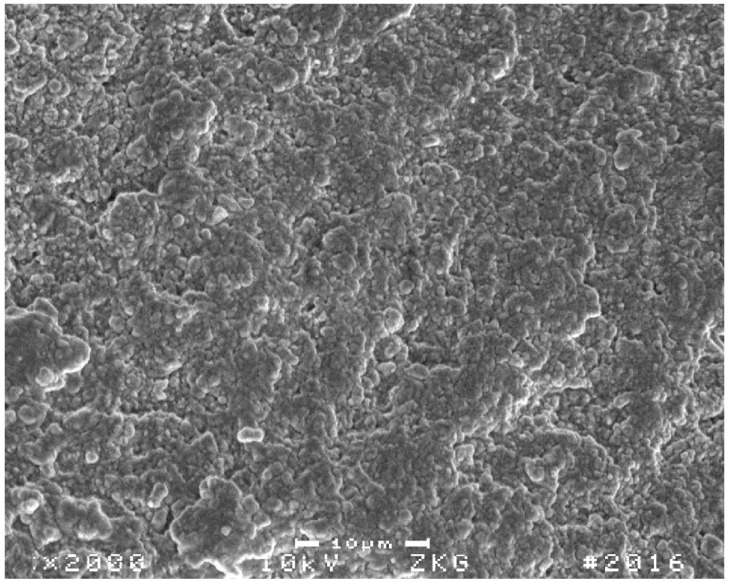
Representative SEM image of a selected TECB fracture surface.

Altogether, a general trend could be observed that showed a lower reliability for the compomer-based restorative materials DY and CGF, which both exhibited only half the high values when compared to the other composite-based materials (Figure 5). In the literature, such markedly different properties are often related to a material’s degree of homogeneity and a deviant flaw size distribution. These have been similarly observed in previous studies reporting Weibull statistics when composite materials were compared with compomers [49,50] or glass-ionomer restoratives [51]. Although, further reasons may exist to explain the diverging results observed for DY and CGF, flaws appear to be the most suitable explanation. Usually flaws, acting as stress concentrators and accordingly influencing the magnitude of the Weibull modulus, are characterised as scratches [36], non-uniform matrix and filler interfaces [52], foreign bodies [53], pores [54], different filler sizes [55,56], and other inherent defects. Such changes in microstructure and the influence on material behaviour may be investigated via Hertzian indentation as previously reported [57,58] where porosity in glass-ionomer cements was examined to gain a better understanding of crack propagation effects [57]. Likewise, in the present study, it may be speculated whether similar pore structures would have significantly contributed to lower m values of DY and CGF, as both materials were delivered in compules compared to TECB and TEC, which were divergently provided in composite syringes. Compules are known to bear a greater risk of incorporated bubbles and so a higher inhomogeneity [59], which both result from the industrial filling processes of compules. Representative SEM images showing different fracture surfaces of bend bars tested in the present study are displayed in Figure 11 for CGF und Figure 12 for TECB. Both images corroborate the above discussed influence of porosities on material properties, showing for CGF markedly higher inhomogeneities in the material structure.

Although SDR was also delivered in compules, a recent study for SDR restorations attested to the lowest amount of porosity when the material was cured [59]. This result is confirmed by the present study, which for SDR shows a Weibull modulus twice as high as those for DY and CGF, which means it more closely resembles the composite restorative material TECB. Of course, other factors might also have contributed to the lower Weibull modulus of DY and CGF, but this needs further clarification.

### 4.3. Damping Parameters

Given all previously discussed common mechanical properties, it can be concluded that VD appears to represent the most resistant and stable material for preparing long-lasting tooth restorations. This assumption is based on the fact that VD has the highest determined values in the cases discussed. Nevertheless, it is not entirely clear whether these properties also adequately outline energy dissipation characteristics and damping behaviour of restorative materials, as these are important characteristics for protecting structures from fracture scenarios in the long term. This is particularly the case if the option of a repeated activation during lifetime, is highly desirable because this attribute is a necessary prerequisite for guaranteeing long-lasting restoration survival. Thus, when looking at the other parameters investigated, namely MT, HLD_dis_ and η_ITdis_, which all three independently characterise damping on their own, it can be stated that in all cases, the situation changes and that instead of VD, SDR always showed the significantly highest values determined in this study (Figure 4, Figure 6 and Figure 10). Given this result and the observation that the damping parameters MT, HLD_dis_, and η_ITdis_ investigated are not independent of the tested restorative materials, the previously formulated null hypothesis must be partly rejected.

The results show that the composite-based material SDR apparently has a particular chemical composition to generate its characteristic material behaviour even though it possesses the lowest filler content of all restorative materials investigated (Table 1). Moreover, SDR exhibited not only the highest damping characteristics but also the second highest FS data in the present study (Figure 2). The ‘Scientific Compendium’ of the manufacturer [60] states that the ‘SDR technology’ has a patented urethane dimethacrylate structure that is not only responsible for a reduction in polymerisation shrinkage [61] and a lower polymerisation stress [62,63] but also comprises a high molecular weight ‘polymerisation modulator chemically embedded in the centre of the polymerisable resin backbone’ which is claimed to be accountable for an optimised viscoelastic behaviour and its resultant material flexibility. These particular material properties, which are likewise responsible for highly desirable energy dissipation effects, provide pronounced damping properties characterised via ‘Dynamic mechanical analysis (DMA)’ by showing high values for tan delta of SDR in the related ‘Scientific Compendium’ [60].

The meaningful parameter ‘tan delta’ and especially its potential correlation to HLD_dis_ data sets, which were likewise determined in the present study, was evaluated and discussed in our previous investigations that examined the damping capacity of common resin-based computer-aided design and computer-aided manufacturing restorative materials [11,12]. As a result of those studies, it was concluded that tan delta data only characterises the viscoelastic part of material damping, not the proportion resulting from plastic deformation. Conversely, plastic deformation, which per definition equally contributes to damping effects [64], was reported to be perfectly captured together with viscoelastic properties via HLD_dis_ data, which therefore, more precisely describes the total damping effect [11]. These previous findings can be transferred to and applied in the present study, where SDR showed the highest HLD_dis_ data (Figure 6) and so pronounced damping effects while concomitantly revealing the highest indentation volume of 2.54 × 10^5^ µm^3^ and indentation depth (2.49 µm) in this study (Figure 7). This discovery, which demonstrated a relatively high degree of plastic deformation for SDR, thus emerging as a damping effect, was unequivocally supported by the results obtained for MT (Figure 4) and η_ITdis_ (Figure 10). These also showed the highest data sets observed for SDR in this study and accordingly verified its high degree of plastic deformation, especially when compared to the compomers DY and CGF. Similarly, in a recent study that investigated the flexural toughness (MT) during 4-point bending for ion-leaching restorative materials, significantly higher values were observed for a microhybrid resin composite used as reference material (2.099 mJ/mm^3^) and resin-based ion-leaching restorative materials like ‘Activa Bioactive Restorative’ (1.880 mJ/mm^3^) compared to ordinary glass-ionomer cements (<0.060 mJ/mm^3^) [50]. Hence, both of these resin-based materials, which are also widely used in paediatric dentistry, largely approach the MT results of SDR (2.98 MJ/m^3^ = 2.98 mJ/mm^3^) and, with that, its damping properties documented in the present study. From a clinical point of view, damping characteristics partially realised via plastic deformation are highly desirable to avoid gaps, micro cracks and imperfect margins, which in a worst-case scenario could result in material failure or restoration loss in the long term.

The positive effect of damping and plastic deformation to avoid microleakage and improve marginal seal may have been the reason for a better performance of SDR on dentin surfaces when compared to VD [65]. Although in the respective study, both restorative materials exhibited similar results on the rather rigid enamel surfaces, a markedly different behaviour was observed on the comparatively tough dentin structures, which generally show a high viscoelastic material behaviour and so more prominent damping characteristics [6]. This different outcome may be explained by the results of the present study, where SDR constantly demonstrated higher damping characteristics and tougher material behaviour than VD and hence a superior ‘property fit’ to the natural viscoelastic dentin structure.

Furthermore, similar energy dissipation characteristics via plastic deformation attenuating destructive energy could also have contributed to the notable material performance previously reported for SDR [66,67,68,69] and SDR/TECB [70]. TECB showed the second deepest indentation volume for the HLD_dis_ determination in the present study (2.09 µm) (Figure 7) and this particular plastic deformation behaviour may also have contributed to the good results for internal gap formation observed for TECB apart from SDR [70]. This higher degree of plastic deformation observed for SDR and TECB was previously found in ‘creep deformation’ investigations where both materials exhibited the highest determined ‘maximum creep strain’ and ‘permanent set’ [71], which further corroborates the findings for SDR and TECB in the present study. In general, the equivalent performance of bulk-filled SDR (4-mm layer) compared to conventionally multilayer-filled restorative materials (2-mm incremental) [72,73,74,75] may in part be attributed to the pronounced damping characteristics explicitly claimed for SDR in the ‘Scientific Compendium’ of the manufacturer [60] and unequivocally substantiated by the results of the present study.

Only a few recent reports from clinical evaluations describe some concerns for SDR. These refer to marginal adaption and marginal discolouration after two [76] and three years [77] performance in class II restorations. However, these results might also have been caused by the final layer of the microhybrid resin composite used that is explicitly recommended by the manufacturer [60] and obviously essential for SDR restorations in clinical practice to efficaciously replace natural enamel structures. Especially this objective fact of a requested different final composite layer represents a limitation of the present study and also, in general, of comparative in vitro and in vivo investigations involving SDR that have been published so far. A reliable comparison of material properties usually involves monolithic materials tested in well-defined set-ups, and material combinations are rarely investigated due to the possible influencing errors of an assembly. Therefore, a meaningful comparison of different restorative materials with the aim of estimating clinical performance is only possible if all essential properties have been separately investigated with monolithic test specimens and are encompassed in the respective assessment. Hence, in many bulk-fill studies, pure composites such as TECB are tested, which do not need a final separate composite layer to replace enamel like SDR restorations do in clinical practice. Therefore, some important material properties like abrasion resistance, occlusal stability and marginal adaption, may not be finally assessed in a comparative manner, as these inherent material characteristics mainly relate to the finally applied composite layer. Consequently, study outcomes obtained with SDR, where SDR was applied in strict accordance with the manufacturer’s recommendations, can only be compared to a limited extend with the results of other bulk-fill composites with monolithic application technique.

Nonetheless, the bulk-fill technique, in general, and especially when applied for the restoration of deciduous teeth, appears to be a suitable alternative to the layering technique due to its ease and speed of application and highly promising clinical outcomes [9,78,79]. This technique is particularly important if patient compliance and the time factor become influencing variables to achieve successful restorations, as is the case for paediatric dentistry. Although SDR restorations need slightly more work input and therefore time, due to a separate step to apply a final and separate enamel restoration layer, compared to common bulk-fill composites, the concept of providing different restorative materials with particular biomimetic material properties to specifically replace natural enamel and dentin structures seems to be highly promising. This approach was already practised perfectly using a CAD/CAM system to precisely mill an extracted third molar, which was subsequently used to successfully restore an extensively damaged tooth [80]. Hence, new technologies like 3D printing [81,82] and sophisticated CAD/CAM restorative materials [83] appear to be very promising building blocks that could facilitate the focused engineering of unique artificial structures with specific mechanical properties and optimally reproduce mother nature’s proven tooth structures on a composite basis. In this context, viscoelastic material properties that provide further improved damping characteristics should focus more on new material development to purposefully replace dentin structures. In addition, highly abrasion-resistant materials should be devised to specifically replace natural robust enamel with optimised biomimetic substitute materials.

## 5. Conclusions

Within the limitations of the present in vitro investigation, the following conclusions can be drawn. For common mechanical properties, Venus Diamond frequently showed the significantly highest data sets and SDR flow the lowest results. In contrast to this outcome, properties describing solely the damping behaviour of restorative materials generally exhibited the significantly highest results for SDR flow and, accordingly, superior capabilities to dissipate harmful fracture energy. The highest degree of plastic deformation, which is also known to be an integral part of a material’s damping characteristics, was found for SDR. Although common mechanical properties do not generally allow a sufficient and meaningful description of damping properties, their characterisation is facilitated by determining the modulus of toughness, HLD_dis_, and η_ITdis_ data. These, therefore, represent new tools for future studies and novel material development.

SDR flow appears to be the most suitable and biomimetic restorative material to rebuild dentin structures showing pronounced viscoelastic properties and, accordingly, superior damping characteristics actually. Future developments in material optimisation should focus on implementing further enhanced viscoelastic material properties to continuously improve energy dissipation characteristics, which allow compensation for the lost damping properties of natural tissues.

## Figures and Tables

**Table 1:** Materials tested.

Material Type	Brand	Code	Manufacturer	Lot No.	Filler Content/wt% *
Compomer	Compoglass F A3	CGF	Ivoclar Vivadent AG (Schaan, Principality of Liechtenstein)	U33209 ^a^	77.0, (76 [15])
	Dyract eXtra A3	DY	DENTSPLY DeTrey GmbH (Konstanz, Germany)	1403000083 ^a^	n.a. (76 [15])
Composite	Venus Diamond A3	VD	Kulzer GmbH (Hanau, Germany)	K010078 ^a^	82.0
	Tetric Evo Ceram A3	TEC	Ivoclar Vivadent AG (Schaan, Principality of Liechtenstein)	Z0067N ^b^	76.0, (73.0 [16])
	Tetric Evo Ceram Bulk Fill A3	TECB	Ivoclar Vivadent AG (Schaan, Principality of Liechtenstein)	Z0038Z ^b^	79–81 ^$^, (73.1 [17])
	SDR flow + U	SDR	DENTSPLY DeTrey GmbH (Konstanz, Germany)	00070112 ^a^	68.0, (69.0 [17]), (67.6 [16])

* Manufacturer information from technical datasheet. ^$^ including 17% prepolymers. n.a., no information available; ^a^ tips application, ^b^ syringe application.

**Table 2:** Mechanical properties of tested materials (selected literature data).

Material Type	Code	FS/MPa *	ME/GPa *	FS/MPa	ME/GPa
Compomer	CGF	110.0 ^a^	8.2 ^a^	67.0 ^b^ [23], 86.9 ^a^ [40], 104.0 ^a^ [41]	17.4 ^c^ [23], 8.8 ^a^ [41]
	DY	118.0 ^a^	7.7 ^a^	78.0 ^b^ [23], 101.0 ^a^ [41]	19.9 ^c^ [23], 7.3 ^a^ [41]
Composite	VD	169.0 ^a^	12.6 ^a^	130.1 ^c^ [34], 157.4 ^a^ [42], 165.3 ^a^ [43]	10.9 ^a^ [42], 7.2 ^a^ [43], 8.7 ^a^ [34]
	TEC	120.0 ^a^	10.0 ^a^	90.6 ^c^ [33], 96.0 ^a^ [41], 107.5 ^a^ [42]	5.3 ^a^ [41], 8.9 ^a^ [42]
	TECB	120.0 ^a^	10.0 ^a^	120.8 ^a^ [44], 87 ^d^ [45]	4.5 ^a^ [44], 9.4 ^d^ [45]
	SDR	118.0 ^a^	5.8 ^a^	131.8 ^a^ [44], 115 ^d^ [45]	5.0 ^a^ [44], 5.9 ^d^ [45]

* Data from manufacturer specification. ^a^ 3-point bending after 24 h, 37 °C, distilled water; ^b^ 4-point bending after 28 d, 37 °C, distilled water; ^c^ 4-point bending after 14 d, 37 °C, distilled water; ^d^ 3-point bending after 48 h, 37 °C, distilled water.

## Data Availability

Not applicable.
